# Analysis of Ecological Environment in the Shanxi Section of the Yellow River Basin and Coal Mining Area Based on Improved Remote Sensing Ecological Index

**DOI:** 10.3390/s24206560

**Published:** 2024-10-11

**Authors:** Huabin Chai, Yuqiao Zhao, Hui Xu, Mingtao Xu, Wanyin Li, Lulu Chen, Zhan Wang

**Affiliations:** School of Surveying and Land Information Engineering, Henan Polytechnic University, Jiaozuo 454000, China; chaihb@hpu.edu.cn (H.C.); 212204020092@home.hpu.edu.cn (H.X.); 212204010035@home.hpu.edu.cn (M.X.); 212304020078@home.hpu.edu.cn (W.L.); 212304020088@home.hpu.edu.cn (L.C.); 212304020098@home.hpu.edu.cn (Z.W.)

**Keywords:** improved remote sensing ecological index, Google Earth Engine, Shanxi section of the Yellow River Basin, coal mining areas, eco-environmental quality

## Abstract

As a major coal-producing area, the Shanxi section of the Yellow River Basin has been significantly affected by coal mining activities in the local ecological environment. Therefore, an in-depth study of the ecological evolution in this region holds great scientific significance and practical value. In this study, the Shanxi section of the Yellow River Basin, including its planned coal mining area, was selected as the research subject. An improved remotely sensed ecological index model (NRSEI) integrating the remotely sensed ecological index (RSEI) and net primary productivity (NPP) of vegetation was constructed utilizing the Google Earth Engine platform. The NRSEI time series data from 2003 to 2022 were calculated, and the Sen + Mann–Kendall analysis method was employed to comprehensively assess the ecological environment quality and its evolutionary trends in the study area. The findings in this paper indicate the following data: (1) The contribution of the first principal component of the NRSEI model is more than 70%, and the average correlation coefficient is higher than 0.79. The model effectively integrates the information of multiple ecological indicators and enhances the applicability of regional ecological environment evaluation. (2) Between 2003 and 2022, the ecological environment quality in the Shanxi section of the Yellow River Basin showed an overall upward trend, with the average NRSEI value experiencing phases of fluctuation, increase, decline, and stabilization. The NRSEI values in non-coal mining areas consistently remained higher than those in coal mining areas. (3) Over 60% of the areas have improved ecological conditions, especially in coal mining areas. (4) The impact of coal mining on the ecological environment is significant within a 6 km radius, while the effects gradually diminish in the 6 to 10 km range. This study not only offers a reliable methodology for evaluating ecological environment quality on a large scale and over a long time series but also holds significant guiding value for the ecological restoration and sustainable development of the Shanxi section of the Yellow River Basin and its coal mining area.

## 1. Introduction

The ecological environment serves as the primary source of material resources essential for human survival and development [1]; however, it is also affected by waste generated from human activities and various other factors [2]. With rapid economic and social development, the adverse effects of human activities on global ecosystems have intensified, significantly impacting regional ecological protection and sustainable development management [3,4]. Therefore, objective, scientific, and timely monitoring and assessment of ecological environment quality have become crucial for effectively evaluating regional ecological status, addressing global changes, and achieving regional sustainable development goals [5].

The Yellow River Basin is an important barrier to ecological security and an important economic zone in China [6]. Its ecological vulnerability and structural contradictions are particularly significant [7]. Shanxi Province, located in the Loess Plateau with its undulating topography and relatively low ground vegetation cover, has serious soil erosion problems and significant sediment runoff, making it one of the regions most severely affected by soil erosion in China [8,9]. As an important province in the Yellow River Basin, Shanxi has a strategic position in the ecological pattern of the basin [10]. Additionally, Shanxi is one of the major coal production bases in China, which is of critical importance to the country’s energy security [11]. The Shanxi section of the Yellow River Basin hosts several national coal-planning mining areas, where the ecological environment is particularly fragile. Long-term, large-scale, and high-intensity exploitation of coal resources has exacerbated issues such as surface soil erosion, vegetation destruction, and ecological degradation [12]. To facilitate the sustainable and high-quality development of the Shanxi section of the Yellow River Basin, it is essential to establish a comprehensive long-term evaluation mechanism for the regional ecological environment. This mechanism will provide a scientific basis for ecological protection and sustainable development, ensuring that the region achieves a balance between economic advancement and environmental conservation.

In recent years, the utilization of remote sensing technology in ecological and environmental monitoring has become increasingly prevalent. Hanqiu Xu proposed the Remote Sensing Ecological Index (RSEI), which is entirely constructed from remotely sensed data [13]. This index utilizes principal component analysis to integrate four indicators—greenness, wetness, dryness, and heat—to monitor spatial and temporal changes in the ecological environment. The four indicators selected are closely related to human survival and are highly representative. A significant advantage of RSEI is that it does not rely on human-set weights of ecological indicators and utilizes the Principal Component Analysis (PCA) method to automatically determine the contribution of each ecological indicator to the principal components, avoiding the errors associated with subjective weight assignments and improving the objectivity and stability of the computational results [14]. Since its introduction, RSEI has been extensively employed in the objective monitoring and evaluation of ecological conditions across various regions and scales due to its advantages of easy data collection, concise process, and visualization of results. Scholars have widely utilized the Remote Sensing Ecological Index (RSEI) to assess ecological conditions in diverse regions. For instance, in urban areas, several studies have demonstrated its effectiveness in characterizing the ecological status [15,16,17,18,19]. In watersheds, a series of research efforts have employed the RSEI to analyze ecological conditions [20,21,22，^23^，^24^]. In the study of forest ecosystems, the RSEI has been employed to quantify changes in forest cover and their impacts on ecosystem services [25]. In wetland ecosystems, the RSEI has successfully elucidated trends in wetland degradation and assessed the effectiveness of conservation measures [26]. Furthermore, in coal mining regions, the RSEI has been utilized to monitor the extent of environmental degradation and to provide decision-making support for ecological restoration efforts [27,28,29,30]. They have examined the spatial distribution, temporal evolution, and influencing factors to offer scientific references and recommendations for ecological restoration and sustainable development in each context. However, RSEI was initially composed of greenness, heat, humidity, and dryness indicators, which only considered four aspects of environmental change without taking into account the geographical characteristics of ecological environments in different regions and anthropogenic disturbances and often failed to achieve the monitoring effect in some regions with more significant characteristics. In view of the above shortcomings, scholars are exploring the improvement methods of RSEI in order to characterize the ecological status of specific regions in a more scientific and reasonable way. According to the characteristics of geographic environment as well as human activity conditions in different study areas, selecting different indicators or adding new indicators on the basis of RSEI for principal component analysis to construct an improved remote sensing ecological index is one of the most common methods to improve RSEI [31,32,33,34,35]. Wang J. et al. [31] constructed the Arid Remote Sensing Ecological Index (ARSEI) by incorporating salinity and land degradation indicators into the principal component analysis in the study of specific arid zones. Zhu D et al. [36] introduced an improved ecological index (MW-RSEI) based on RSEI and moving window evaluation unit and applied it to the evaluation of ecological environment in mining area, considering the difference in ecological impact range of different damaged characteristic land in mining area (such as mining field, subsidence area, dump, etc.). These studies demonstrate the necessity and importance of improving and optimizing the remote sensing ecological index in a specific ecological environment.

Despite numerous studies on ecological changes, there is still a lack of targeted assessment models for the specific area of the Shanxi section of the Yellow River Basin, particularly regarding the complex ecological challenges brought about by coal mining. Therefore, this study proposes an improved remote sensing ecological index (NRSEI), which combines net primary productivity (NPP) with key ecological factors such as greenness, humidity, dryness, and heat, to systematically analyze the ecological environment changes in this region. Compared to traditional methods, NRSEI can more accurately reflect the ecological health status of the area, showcasing its unique advantages in assessing the impact of coal mining on the ecological environment. Moreover, this research provides a scientific basis for developing more effective ecological restoration policies, thereby promoting sustainable development in the region.

## 2. Materials and Methods

### 2.1. Study Area

**Figure 1:**
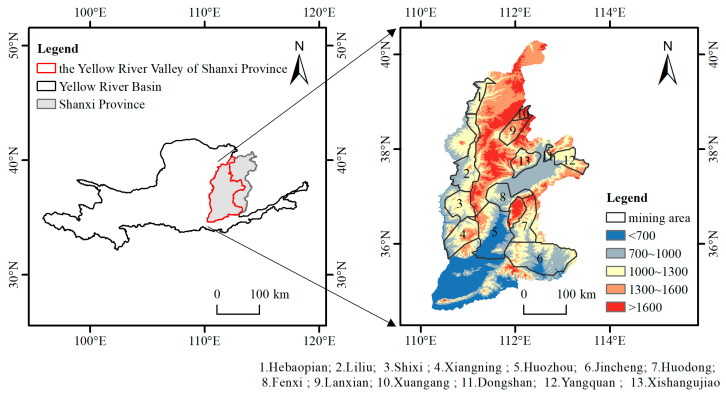
Overview of the study area.

The Shanxi section of the Yellow River (34°33′~40°19′ N, 110°12′~113°38′ E) is a crucial component of the middle reaches of the Yellow River. The main stream of the Yellow River enters Shanxi from Pianguan County, flowing through 11 regions, including Shuozhou and Xinzhou, and exits the province at Yuanqu’s Nianpangou. The basin area of this region spans approximately 97,100 square kilometers, representing 62.2% of Shanxi Province’s total area, making it a significant water resource and ecological treasure trove for the province. The study area is mainly composed of Loess Plateau mountain area; the terrain is undulating, and the landform is complex and diverse. In the western region, the Lvliang Mountains and Loess Hills are characterized by abundant forest resources, albeit of low quality, and are significantly affected by soil erosion. The central area features flat terrain with a favorable environment, while the eastern region presents variable topography and a fragile ecological environment. Furthermore, the Shanxi section of the Yellow River Basin is endowed with rich coal resources, encompassing 13 national coal mining areas (as shown in Figure 1). However, these areas also possess relatively fragile natural ecosystems that are highly vulnerable to human activities. The extraction of coal resources has resulted in irreversible negative impacts on the regional ecological environment and has intensified the process of ecological degradation.

### 2.2. Data Sources and Preprocessing

All remote sensing data employed in this study were procured from the Google Earth Engine cloud platform (https://earthengine.google.org, which was accessed on 30 June 2024), as delineated in Table 1. MODIS, provided by the National Aeronautics and Space Administration (NASA), is an advanced remote sensing sensor mounted on the Terra and Aqua satellites. It features high temporal resolution, high spatial resolution, and a vast accumulation of historical data, making it particularly suitable for monitoring ecological conditions in both the past and present [37,38]. To reduce the uncertainty caused by seasonal variations, this study selected MODIS data from the vegetation growing season (June to September) during the period from 2003 to 2022 to monitor the ecological changes and evolutionary trends in the Shanxi section of the Yellow River Basin. Leveraging the powerful Google Earth Engine cloud computing platform, the NDVI greenness indicator was extracted from the MOD13A1 image set, the time series LST image of temperature index is extracted from the MOD11A2 image set, and the heat and dryness indicators were extracted from the surface reflectance data of MOD09A1 products. The Moderate Resolution Image Spectroradiometer (MODIS) reflectance products (MOD09A1 and MYDO9A1) were selected to compute a number of vegetation-related indices (including NDVI, NDWI, NDBI, LSWI, NIRV, and SR) on Google Earth Engine, and the meteorological data were extracted from the Terra Climate dataset using the MODIS annual land cover type product (MCD12Q1) to calculate the cumulative number of land use changes in each pixel during the study period, which was used to further calculate NPP and construct the model. All remotely sensed data were resampled to 500 m, and the projected coordinate system was WGS_1984_Word_Mercator.

The rest of the data, including China Digital Elevation Model (DEM) data and administrative boundary data, were obtained from the Resource and Environment Data Center of the Chinese Academy of Sciences (https://www.resdc.cn/, which was accessed on 10 June 2024).

### 2.3. Research Methods

#### 2.3.1. NRSEI Component Indicator Calculation

This study builds upon the RSEI evaluation method by incorporating Net Primary Productivity (NPP) as a carbon sink component. An improved Remote Sensing Ecological Index (NRSEI) is constructed using principal component analysis. This method aims to reflect the ecological quality of the Shanxi section of the Yellow River Basin and the coal mining area more scientifically and objectively. Specifically, the normalized difference vegetation index (NDVI) is extracted from remote sensing images to represent greenness. Land surface temperature is derived from thermal infrared bands to indicate heat. A cap transformation calculates the humidity index, while the normalized difference built-up-soil index represents dryness. Meanwhile, the CASA model is used to compute the NPP index.

(1) Greenness Indicator

The normalized difference vegetation index was selected to represent the greenness indicator [39]. It effectively characterizes the growth and nutritional status of surface vegetation and is closely related to plant biomass, leaf area index, and vegetation coverage [40]. The calculation formula is as follows:

(1) $$NDVI=M_{NDVI}\times 0.0001$$

where NDVI is the Normalized Difference Vegetation Index and $M_{NDVI}$ is the value of the NDVI band in the MOD13A1 dataset.

(2) Wetness Indicator

The wetness component was selected to represent the wetness indicator. The wetness index from the tasseled cap transformation is closely related to the moisture content of vegetation and soil [41]. The formula is as follows:

(2) $$WET=0.1147\rho_{red}+0.2489\rho_{NIR1}+0.2048\rho_{\mathrm{bl}ue}+0.3132\rho_{green}-0.3122\rho_{NIR2}-0.6416\rho_{SWIR1}-0.5087\rho_{SWIR2}$$

where WET is the humidity component of tasseled cap transformation for MODIS data and $\rho_{red}$, $\rho_{NIR1}$, $\rho_{blue}$, $\rho_{green}$, $\rho_{NIR2}$, $\rho_{SWIR1}$, and $\rho_{SWIR2}$ are the red, near infrared 1, blue, green, near infrared 2, short infrared 1, and short infrared 2 of the MOD09A1 dataset, respectively.

(3) Heat Indicator

In this paper, land surface temperature is selected to represent the heat index. Land surface temperature is an important parameter in the surface energy balance, which effectively represents the degree of water and energy exchange between the surface and the atmosphere [42]. The calculation formula is as follows:

(3) $$LST=M_{LST}\times 0.02-273.15$$

where LST is the surface temperature and the unit is °C; it is the value of LST _ Day _ 1km band of MOD11A2 dataset.

(4) Dryness Indicator

The dryness index is used to represent the dryness index, that is, the ‘dry’ degree of the surface. This index is not the opposite of the humidity index; it also includes the degree of hardening of the ground. The index-based built-up area index (IBI) and soil index (SI) represent the surface physical state of the built-up area and bare land, respectively. The normalized difference building-soil index is obtained by combining the average values, which can effectively characterize the drought degree of the surface and is suitable for the mountainous terrain in the Shanxi section of the Yellow River Basin. The calculation formula is as follows:

(4) $$NDBSI=\frac{IBI+SI}{2}$$

(5) $$IBI=\frac{2\rho_{SWIR1}/(\rho_{SWIR1}+\rho_{NIR1})-\left(\frac{\rho_{NIR1}}{\rho_{NIR1}+\rho_{\mathrm{red}}}+\frac{\rho_{green}}{\rho_{green}+\rho_{NIR1}}\right)}{2\rho_{SWIR1}/(\rho_{SWIR1}+\rho_{NIR1})+\left(\frac{\rho_{NIR1}}{\rho_{NIR1}+\rho_{\mathrm{red}}}+\frac{\rho_{green}}{\rho_{green}+\rho_{NIR1}}\right)}$$

(6) $$SI=\left(\rho_{SWIR1}+\rho_{red}\right)-\left(\rho_{blue}+\rho_{NIR1}\right)/\left(\rho_{SWIR1}+\rho_{red}\right)+\left(\rho_{blue}+\rho_{NIR1}\right)$$

where NDBSI is the building and bare soil index, IBI is the construction index, and SI is the bare soil index.

(5) NPP Indicator

As a pivotal component of the surface carbon cycle, the net primary productivity (NPP)) of vegetation is an important indicator to characterize carbon balance, nutrient cycling, and the sustainable development of terrestrial ecosystems. Therefore, it can be used as an important parameter to reflect the damage of ecological pollution [43]. The Carnegie–Ames–Stanford Approach (CASA) model is a satellite-based model of photosynthetic utilization that has been widely used in the calculation of NPP at the regional scale [44]. Unlike many studies that utilize the MOD17A3HGF dataset to calculate net primary productivity (NPP), this study employs the Carnegie–Ames–Stanford Approach (CASA) model for NPP assessment. This methodology has been validated and applied in various contexts, including carbon research and ecological change [35,36]. The calculation formula for NPP in the CASA model is as follows:

(7) NPP(x,t)=APAR(x,t)×ε(x,t)

where APAR(x,t) represents the photosynthetically active radiation (MJ/m^2^) absorbed by pixel *x* in *t* month and ε(x,t) represents the light energy utilization rate of pixel *x* in month *t*.

(8) APAR(x,t)=r×SOL(x,t)×FPAR(x,t)

where SOL(x,t) represents the total solar radiation absorbed by pixel *x* in month t andFPAR(x,t) indicates the photosynthetically active radiation of pixel *x* in *t* month. Constant r≈0.5 represents the available solar effective radiation ratio of vegetation, that is, the ratio of FRAR to SOL.

(9) $$\varepsilon (x,t)=T_{\varepsilon 1}(x,t)\times T_{\varepsilon 2}(x,t)\times W_{\varepsilon }(x,t)\times \varepsilon_{\max}$$

where $T_{\varepsilon 1}(x,t)$ and $T_{\varepsilon 2}(x,t)$ represents the influence of low temperature and high temperature on the light energy utilization rate of pixel *x* in *t* month, respectively; $W_{\varepsilon }(x,t)$ represents the influence of precipitation on the light energy utilization rate of pixel *x* in *t* month; and $\varepsilon_{\max}$ represents the maximum light energy utilization rate of vegetation.

#### 2.3.2. NRSEI Model Construction

The presence of a large volume of water body information affects the results of NRSEI calculations, so the regional water bodies need to be masked using the Modified Normalized Difference Water Index (MNDWI) before constructing the model. The calculation formula is as follows:

(10) $$MNDWI=\frac{\rho_{green}-\rho_{SWIR1}}{\rho_{green}+\rho_{SWIR1}}$$

where $\rho_{green}$ and $\rho_{SWIR1}$ represent the band values of green and shortwave infrared 1 in the multispectral data, respectively.

Before using principal component analysis to integrate the five indicators of greenness, humidity, heat, dryness, and carbon sink component, due to the differences in units and sizes of each indicator component, in order to avoid the weight imbalance caused by the inconsistency of the scale, it is necessary to normalize the five indicators, and the normalization calculation formula is as follows:

(11) $$NI_{i}=I_{i}-I_{min}/I_{max}-I_{min}$$

where $NI_{i}$ is the normalized value of a certain index value in the pixel i, $I_{i}$ is the value of the index in the pixel, $I_{max}$ is the maximum value of the index, and $I_{min}$ is the minimum value of the index.

To further confirm the weights of each of the standardized five index components in the composite index NRSEI, principal component analysis was used to calculate the weights. In this study, the first principal component was extracted to construct the improved remote sensing ecological index (NRSEI). In the results of principal component analysis, PC1 was subtracted by 1 only when the loadings of greenness (NDVI), humidity (WET), and net primary productivity (NPP), which play a positive role in ecology, were negative in PC1, as represented in the following equation:

(12) $$NRSEI_{0}=\left\{\begin{array}{l} PC_{1}\left[NDVI,\;WET,\;NPP,\;LST,\;NDBSI\right],\;NDVI>0\;and\;WET>0\;and\;NPP>0\\ 1-PC_{1}\left[NDVI,WET,NPP,LST,NDBSI\right],\;NDVI<0\;and\;WET<0\;and\;NPP<0 \end{array}\right.$$

where $PC_{1}$ is the first principal component and $NRSEI_{0}$ denotes the initial NRSEI.

In order to facilitate the measurement and comparison of the indicators, the initial improved remote sensing ecological index is normalized so that its value lies in the interval of [0,1] to obtain the *NRSEI*, which is calculated by the following formula:

(13) $$NRSEI=\frac{NRSEI_{0}-NRSEI_{0\_min}}{NRSEI_{0\_max}-NRSEI_{0\_min}}$$

where $NRSEI_{0}$ denotes the initial improved remote sensing ecological index, $NRSEI_{0\_min}$ denotes the minimum value of NRSEI, and $NRSEI_{0\_max}$ denotes the maximum value of NRSEI. When the value of NRSEI is closer to 1, it means the ecological environment quality is better; when the value of NRSEI is closer to 0, it means the ecological environment quality is worse. In order to visualize and quantitatively assess the ecological environmental quality of the Shanxi section of the Yellow River Basin and coal mining areas, this paper adopts the equally spaced grading method to divide the NRSEI into five grades with 0.2 as the boundary, as shown in Table 2.

#### 2.3.3. Sen + Mann–Kendall Trend Analysis

The Sen slope estimator is a robust non-parametric statistical method for estimating the trend of a time series [45,46]. The method is computationally efficient and insensitive to outliers, thus making it suitable for long-term trend analysis of ecological indicators. The Mann–Kendall significance test is a non-parametric statistical method that does not require the sample to follow a normal distribution and handles missing values and outliers well, making it suitable for assessing pivotal components of ecological quality over time [47]. Theil–Sen and Mann–Kendall’s combined method is a common method for estimating the trend of a time series and determining its significance, which is calculated as follows:

(14) $$\beta =Median\left(\frac{x_{j}-x_{i}}{j-i}\right),\forall j>i,$$

where $x_{i}$ and $x_{j}$ represent the number of time series and β is the Sen slope estimator of the NRSEI time series trend. The positive β value indicates that the time series is on the rise, and the negative value indicates that the time is on the decline. Median is a median function; i and j are the number of time series, respectively; $x_{i}$ and $x_{j}$ are the NRSEI values at moments i and j, respectively.

(15) $$\theta =x_{j}-x_{i}$$

(16) $$S=\sum_{i=1}^{n-1}\sum_{j=i+1}^{n}\mathrm{sgn}(\theta )$$

where when θ>0, sgn(θ)=1; when θ=0, sgn(θ)=0. Accordingly, we can construct the variance Var(S) of S, the formula used is as follows:

(17) $$Var(S)=[n(n-1)(2n+5)-\sum_{i=1}^{m}t_{i}(t_{i}-1)(2t_{i}+5)]/18$$

where n represents the number of points, m represents the number of groups with the same value of samples, and $t_{i}$ represents the number of parallels in the range i. If n>10, the slope can be expressed by Z, and the calculation formula is as follows:

(18) $$Z=\left\{\begin{array}{l} \left(S-1)/\sqrt{\mathrm{var}(S)},(S>0\right)\\ 0,(S=0)\\ \left(S+1)/\sqrt{\mathrm{var}(S)},(S<0\right) \end{array}\right.$$

The Sen + Mann–Kendall trend analysis uses the parameter β in the Theil–Sen method to determine the direction of the trend and the value of Z in the Mann–Kendall method to assess the significance of the trend. When the significance level b is 0.05, the critical value *Z* is 1.96. To better illustrate the usefulness of the Sen + Mann–Kendall trend analysis approach to ecological quality assessment, Table 3 provides a classification of ecological quality trends based on the slope estimate β and the standardized test statistic |Z|.

## 3. Results

### 3.1. Rationalization Analysis of the Improved NRSEI Model

#### 3.1.1. Principal Component Analysis of the NRSEI Model

The results of principal component analysis (Table 4) show that PC1 has a contribution rate of over 70.28% in each year in the Shanxi section of the Yellow River Basin. The multi-year average value is 75.87%. This indicates that PC1 of the NRSEI model incorporates the main characteristics of the five chosen indicators and is more effective in reflecting the ecological and environmental quality in the study area compared to the other components.

**Figure 2:**
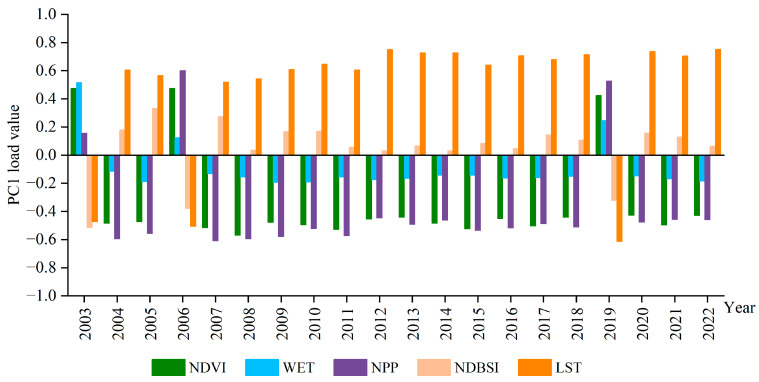
Results of principal component analysis from 2003 to 2022.

The results of the principal components analysis for the indicators of the Natural Resources and Sustainable Environment Index (NRSEI) in the Shanxi section of the Yellow River Basin are presented in Figure 2. The principal component loadings of NDVI, WET, and NPP, which represent greenness, humidity, and carbon sinks, were positive in 2003, 2006, and 2019. This indicates that they had a positive influence on the ecological environment. Conversely, the principal component loadings of heat and dryness were negative, suggesting a negative impact. These findings align with the actual conditions. In the other years, the situation of the principal components’ corresponding coefficients was opposite to that of 2003, 2006, and 2019, thus requiring “1-PC1” treatment, resulting in positive values for NDVI, WET, and NPP, which play a positive role in ecological assessment, while negative values for NDBSI and LST, which play a negative role in ecological quality assessment. The results were that NDVI, WET, and NPP all had positive values, playing a positive role in ecological assessment, while NDBSI and LST all had negative values, playing a negative role in ecological quality assessment.

#### 3.1.2. Comparative Analysis of NRSEI and RSEI

**Figure 3:**
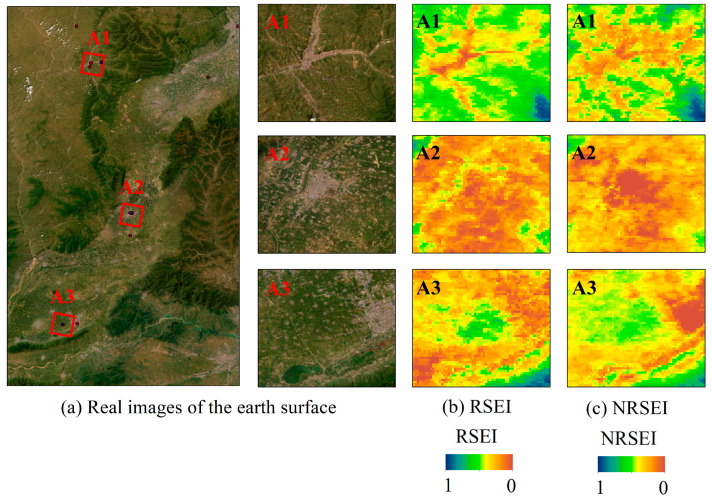
Comparison of regional RSEI and NRSEI results in 2022.

To verify the applicability of NRSEI, three representative areas were selected for comparative analysis, reflecting the characteristics of the Shanxi section of the Yellow River Basin. These areas, A1, A2, and A3, correspond to mine ecological restoration zones located near Lvliang, Linfen, and the Yellow River Wetland Reserve, respectively. The comparative performance of the two indices, RSEI and NRSEI, in 2022 for these typical regions is illustrated in Figure 3. Based on the differences in local details and key areas, NRSEI demonstrates richer spatial information and finer texture details compared to RSEI, aligning more closely with the actual ground cover conditions.

In further analyzing the difference between the application effects of RSEI and NRSEI, information entropy is adopted for quantitative comparative analysis of local detail information. Information entropy can be used to measure the amount of information in an image, and when the entropy value increases, it indicates that the complexity of the image is higher and it contains more information. The information entropy of RSEI and NRSEI is 5.99 and 6.67 for the typical areas selected in Figure 3; for the whole Shanxi section of the Yellow River Basin, the information entropy of RSEI and NRSEI is 4.78 and 4.91, respectively, which shows that, compared with the RSEI, the complexity of the NRSEI image is higher, and the amount of information contained in the NRSEI image is greater, and better reflects the complexity and information of the Yellow River Basin and the planned mines in Shanxi. The results show that compared with RSEI, NRSEI images are more complex, contain more information, and better reflect the real ecological situation of the Shanxi section of the Yellow River Basin and the planned mining area. In summary, NRSEI can effectively integrate the newly added NPP factors, which enhances its applicability in monitoring the ecological environment in ecologically fragile areas and ecological restoration areas.

**Figure 4:**
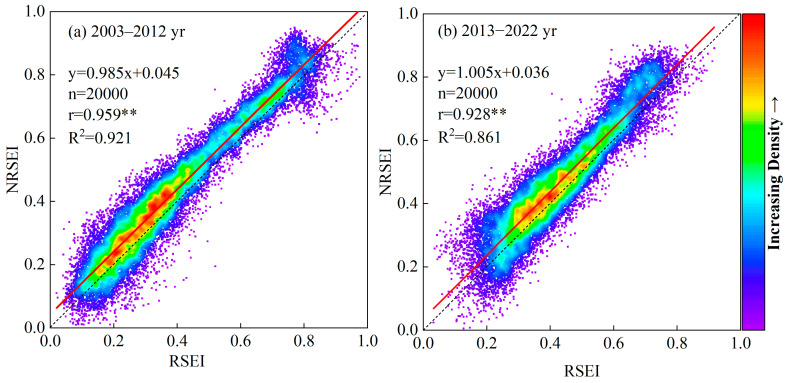
Scatterplot of RSEI and NRSEI. The asterisks (**) indicate a statistically significant correlation at the 0.01 level (two-tailed), suggesting a strong positive relationship between the variables analyzed.

In this study, to further validate the reliability of the NRSEI index model, 20,000 random points were selected across the study area, and the corresponding pixel values were extracted. The mean values of the RSEI and NRSEI results for the periods 2003–2012 and 2013–2022 were calculated, and scatter plots of pixel mean density for both time frames were constructed (Figure 4). The scatter plot for the period of 2003–2012 (Figure 4a) demonstrates a strong correlation between RSEI and NRSEI, with a Pearson correlation coefficient of 0.959 and the R2 value of 0.921, indicating a highly significant linear relationship. For the period of 2013–2022 (Figure 4b), while the correlation between RSEI and NRSEI remains significant, the Pearson correlation coefficient decreases to 0.928, and the R² value drops to 0.861, reflecting a diminished model fit compared to the previous period. A significance test confirmed that the correlation between these datasets reached a statistically significant level (*p* < 0.01), further reinforcing the model’s reliability. The distribution of scatter clusters shows a greater prevalence of outliers on the RSEI side, suggesting that the NRSEI-based model demonstrates greater stability over the entire study period in comparison to the RSEI model. Based on the difference between the 1:1 line and the fitted line, it is clear that the RSEI index is underestimated.

#### 3.1.3. Correlation Test of NRSEI 

Correlation is a quantitative measure of the degree of association between components, and the suitability of the NRSEI model is assessed by calculating the correlation between the indicators. The results of the test of the average correlation between the five ecological indicators selected for the period of 2003–2022 and the NRSEI model are shown in Table 5. The average correlation ranges from 0 to 1. The closer the value is to 1, the greater the correlation, and conversely, the lower the correlation. From the perspective of each index, the average correlation between the indexes is around 0.6; the highest is NDBSI, with an average correlation value of 0.680; and the lowest is WET, with an average correlation value of 0.655. The factor with the highest average correlation is NRSEI, with an average value of 0.804 over a period of 20 years. This average correlation value is greater than that of the other factors. Additionally, the average correlation value of NRSEI reached its maximum value of 0.860 in the year 2005. In 2005, the average correlation of NRSEI reached its peak value of 0.860. The enhanced remote sensing ecological index is evident in its ability to not only combine the information from each individual index but also provide a more representative measure than the individual indexes and better represent the comprehensive ecological status of the region.

### 3.2. Analysis of Habitat Status in the Shanxi Section of the Yellow River Basin and Coal Mining Area

**Figure 5:**
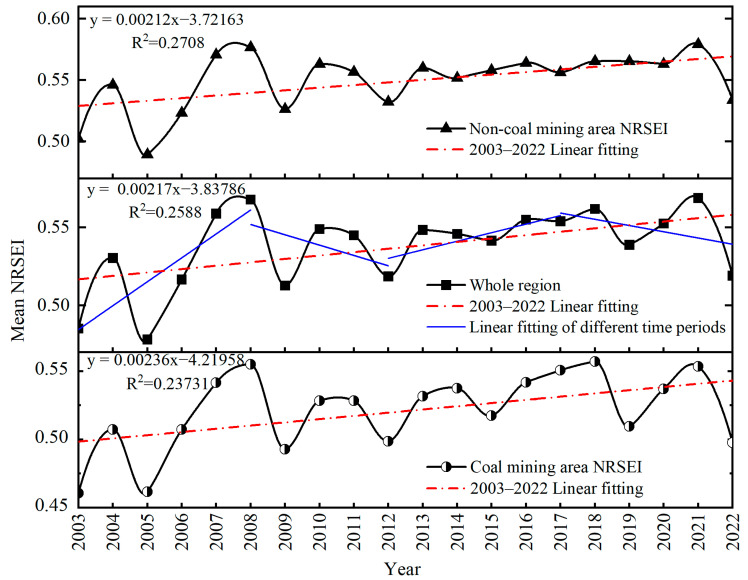
The annual average NRSEI change trend chart of Shanxi Yellow River Basin, planned coal mining area, and non-coal mining area from 2000 to 2022.

The trend of the yearly average NRSEI values of the Yellow River Basin and the planned coal and non-coal mining areas in Shanxi from 2003 to 2022 is shown in Figure 5. It shows that the yearly average NRSEI values of the non-coal mining areas in the consecutive 20 years are higher than those of the entire Shanxi section of the Yellow River Basin, while the coal mining areas have the lowest NRSEI values. The ecological environment evolution process in the Shanxi sector of the Yellow River Basin, including the planned coal mining region and the non-coal mining area, may be categorized into four distinct phases. These phases are demarcated by the years 2008, 2012, and 2017, respectively. ① The period from 2003 to 2008 was a period of fluctuating increase; the average NRSEI value experienced a significant decrease in 2005 and then quickly rebounded, and the ecological environmental quality reached its highest value in 2008. During this period, the NRSEI experienced both increases and decreases, but the overall trend was extremely rapid. The average NRSEI value for the entire study area increased from 0.485 in 2003 to 0.568 in 2008. For non-coal mines, the value increased from 0.502 in 2003 to 0.577 in 2008, and for coal mines, it increased from 0.461 in 2003 to 0.555 in 2008. ② The period from 2008 to 2012 was a period of fluctuating decline. The average NRSEI experienced an increase in 2010, followed by a slow decline, and the average NRSEI value showed an overall decreasing trend during this period, with the lowest value being reached in 2009. ③ The period from 2012 to 2017 was a period of steady rise, a period in which the NRSEI was more stable, with small fluctuations, small standard deviations, and an overall slow upward trend. ④ Fluctuating decline again from 2017 to 2022, a sharp decline in 2019 followed by a slow rise, reaching the highest value of NRSEI in 2021, in which the value of NRSEI for the entire Shanxi section of the Yellow River Basin was 0.569 and the value of NRSEI for the coal mining area was 0.551. NRSEI declined again in 2022. Generally speaking, the ecological environment of the entire Shanxi section of the Yellow River Basin area has slightly improved since 2003–2022. In the study area, non-coal mining areas showed the greatest degree of improvement, and the ecological environment of coal mining areas showed the least degree of improvement.

**Figure 6:**
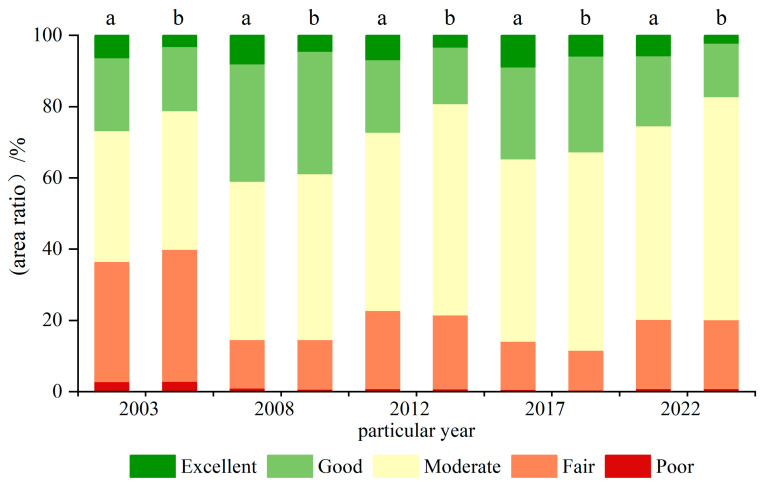
Percentage of area in different ecological classes: (a) the whole region of the Shanxi section of the Yellow River Basin and (b) coal mining area in the Shanxi section of the Yellow River Basin.

In this paper, five specific years, 2003, 2008, 2012, 2017, and 2022, are selected as key points to analyze the NRSEI distribution of the Shanxi section of the Yellow River Basin and its coal mining area in these five years. In order to clarify the ecological environment quality status of various regions, the vector boundaries of the Shanxi section of the Yellow River Basin and the coal mining area were taken as the scope, respectively, and the percentage of the area of each ecological grade in the Shanxi section of the Yellow River Basin and its coal mining area was counted, as illustrated in Figure 6. At the scale of the entire Shanxi section of the Yellow River Basin, the area of areas with “poor” and “fair” ecological grades decreased by 1.93% and 14.27%, respectively. The remaining ecological classes have experienced an expansion in their respective areas, with the “moderate” ecological class exhibiting the most significant rise at 14.27%. At the mine scale, the ecological quality distribution of coal mining regions each year closely matched that of the entire region, with “fair” and “moderate” being the dominant categories. The percentage of regions classified as “poor” and “fair” in terms of ecological quality declined dramatically, by 2.09% and 17.68%, respectively. Meanwhile, the percentage of areas classified as “moderate” increased by 23.73%. The remaining two ecological classifications experienced minimal changes in area. Compared with the entire Shanxi section of the Yellow River Basin region, the ecological quality of coal mining areas in the region is relatively poor, but the gap between the two is gradually narrowing.

**Figure 7:**
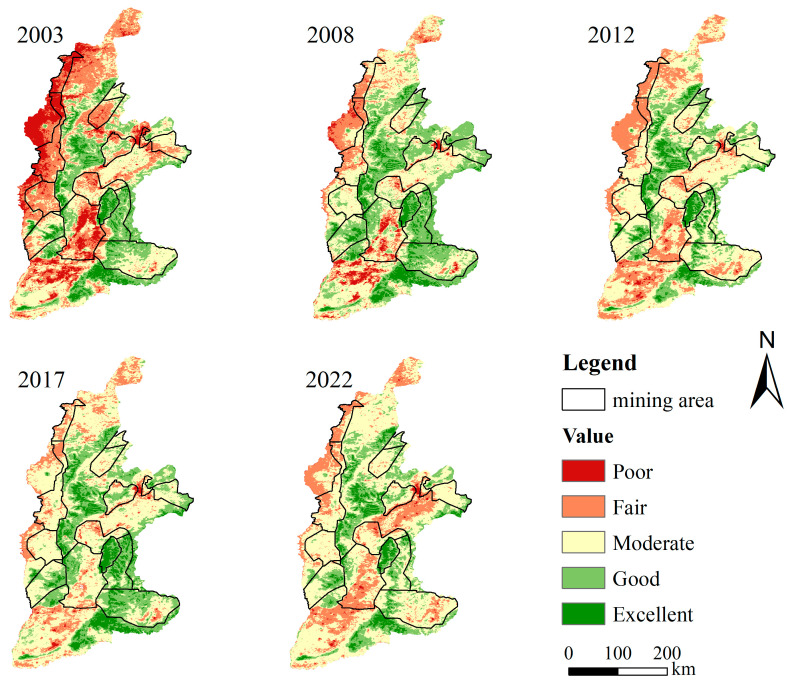
Changes of ecological quality in the Shanxi section of the Yellow River Basin.

Based on the spatial distribution of NRSEI shown in Figure 7, the ecological quality of the Shanxi section of the Yellow River Basin exhibits a spatial pattern characterized by higher quality in the southeast and lower quality in the northwest. The western area between the Yellow River and the Lvliang Mountains, the Linfen Basin, and the Yuncheng Basin are areas of poor ecological quality. The Luliang Mountains and the Taiyue Mountains are ecologically sound areas. In terms of the distribution of NRSEI in coal mining areas, the largest areas are in the categories of “poor”, “poor”, and “fair”. The area of the areas with grades of “poor” and “fair” is larger than the area of the areas with grades of “excellent” and “good”, indicating that the ecological environment quality of the coal mining area is poor. Hebaopian, Liuli, Shixi, and Huozhou mining areas are the coal mining areas with the worst ecological quality, Xiangning, Fenxi, Dongshan, Xishangujiao, and Lanxian mining areas have poorer ecological quality, while Jincheng, Xuangang, Huodong, and Yangquan mining areas have a better ecological quality as a whole.

### 3.3. Dynamic Changes of Ecological Environment in the Shanxi Section of the Yellow River Basin and Coal Mining Area

**Figure 8:**
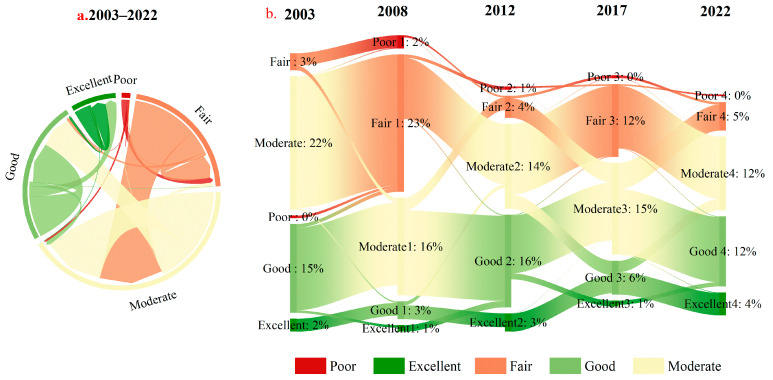
The change in ecological environment quality in the Shanxi section and mining area of the Yellow River Basin. (**a**) The chord plot represents the change in ecological quality over the period 2003–2022. The link is on the area that transitions from one type to another. (**b**) The Sankey diagram represents changes in ecological quality over four time periods. The data on the link represents some of the larger areas that have transitioned from ecological quality.

The dynamic change of NRSEI level in the study area from 2003 to 2022 was further analyzed by using the ecological quality transfer matrix, chord diagram, and Sankey diagram (Figure 8). The results of this study showed that the degree of change in ecological quality was mainly one level in each type of ecological quality level shift. Amongst all, the type of improvement is characterized by “fair” to “moderate”, “moderate” to “good”, and “good” to “excellent”. The ecological grade from “fair” to “moderate” is the largest, accounting for 24.06%, while the remaining two types of change account for 12.21% and 4.01%, respectively. The degradation types are mainly “good” to “moderate” and “moderate” to “fair”, with a percentage of 3.54% and 2.41%, respectively. In terms of different periods, the degree of degradation of the ecological grades was greater than the degree of improvement from 2003 to 2008, and the largest change was from “moderate” to “fair”, which accounted for 22% of the total; the degree of improvement was greater than the degree of degradation in the ecological grades from 2008 to 2012, and the largest change was from “fair” to “moderate”, which accounted for 23%; the largest change in ecological grades from 2012 to 2017 was from “good” to “moderate”, which accounted for 16%; the largest change in ecological rating between 2017 and 2022 is from “moderate” to “good”, with a percentage of 15 percent. Overall, about 51.96% of the areas experienced a change in classification during the study period.

**Figure 9:**
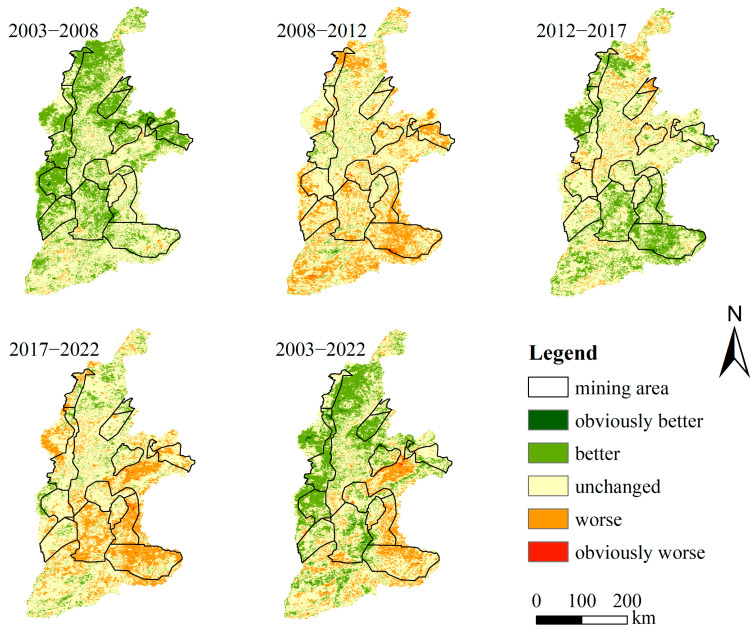
Monitoring of ecological environment quality changes in the Shanxi section and mining area of the Yellow River Basin.

According to the degree of rise and fall of the grade value, the type of change is categorized into five types, namely, obviously worse (−2, −3, −4), worse (−1), unchanged (0), better (+1), and obviously better (+2, +3, +4), and the distribution is shown in Figure 9. In terms of different periods of change, in 2003–2008 and 2012–2017, the green change for the better was greater than the red change for the worse, and the ecological environment improved. In 2008–2012 and 2017–2022, the red change for the worse was greater than the green change for the better, and the ecological environment showed a slight deterioration, which is in line with reality. Overall, the ecological environment quality of the Shanxi section of the Yellow River Basin improved from 2003 to 2022.

### 3.4. The Evolution Trend of Ecological Quality in the Shanxi Yellow River Basin and Coal Mining Area

**Figure 10:**
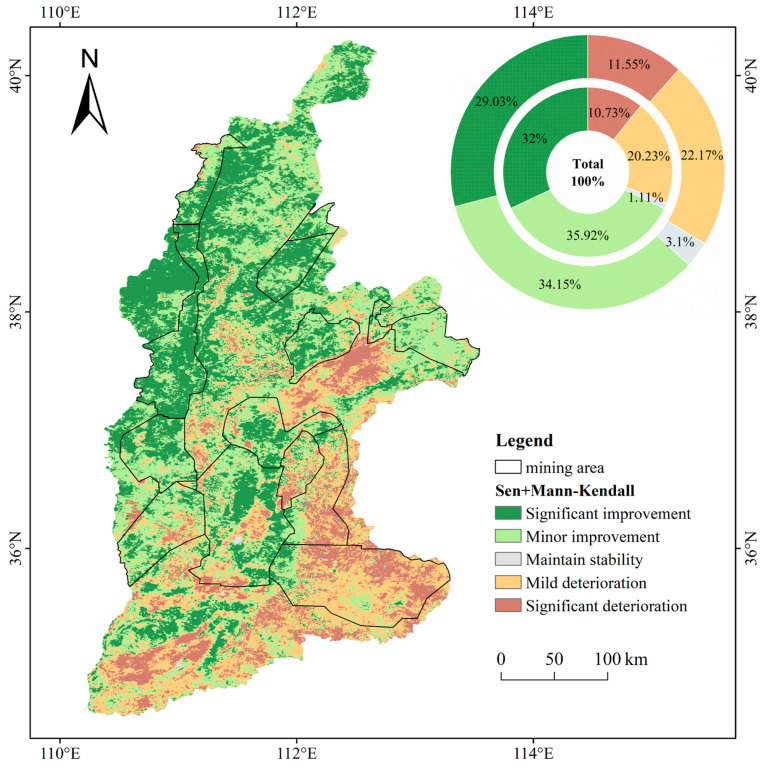
Spatial pattern of remote sensing ecological index change in the Shanxi Yellow River Basin and planned mining area.

To visualize the spatial changes in the ecological environment of the Yellow River Basin and planned mining areas in Shanxi during the study period, we analyzed the spatial changes in the NRSEI using the Sen + Mann–Kendall analysis method. We then categorized the results and calculated the proportions of each change category, as depicted in Figure 10. The results of this study showed that the evolutionary trend of NRSEI is increasing both regionally and in most parts of the coal mining area and passes the test of significance. At the regional scale of the entire Shanxi section of the Yellow River Basin, more than 60% of the area’s ecological environment has been improved between 2003 and 2022. Specifically, 29.03% were categorized as significant improvement and 34.15% were categorized as minor improvement. In contrast, 22.17% of the districts showed mild deterioration, and 11.55% showed significant deterioration. At the coal mine scale, more than 65% of the areas demonstrated ecological improvement. Of these areas, 32% were categorized as significantly improved and 35.92% were categorized as minor improvements; in comparison, 10.73% of the areas showed significant deterioration and 20.23% of the areas showed mild deterioration. In general, the degree of improvement in the mining area is greater than that in the entire region of the Shanxi section of the Yellow River Basin.

In terms of spatial distribution, the areas in the Shanxi section of the Yellow River Basin that show a decrease in the evolutionary trends of NRSEI are primarily concentrated in the basin area. Specifically, there are numerous areas with decreasing evolutionary trends in the Taiyuan, Jincheng, and Yuncheng basins. The evolutionary trend in the southwestern part of the study area is mostly elevated. Among the coal mining areas, the ecological evolution trends of Jincheng, Huodong, and Xuangang mines are all decreasing, while the rest of the mines have a large number of increasing ecological evolution trends.

### 3.5. Ecological Radiation Effect of Mining Area

**Figure 11:**
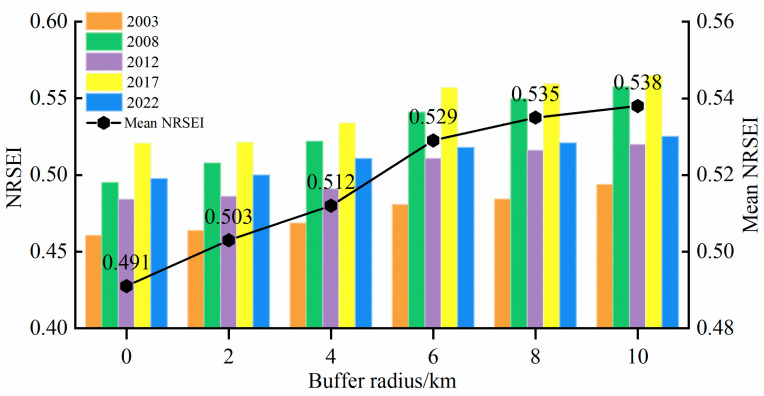
Distribution of NRSEI means in different impact zones.

As shown in Figure 11, in order to explore the impact of coal mining on the ecological environment of the surrounding areas of the mine, five buffer zones were set up at intervals of 2 km outside the planned mine in the Shanxi section of the Yellow River Basin. The total area of the impact zone accounts for 62.35% of the area of the Shanxi section of the Yellow River Basin, which is highly representative. The mean NRSEI values of the impact zones from the inside to the outside obviously show an increasing trend, and when the buffer zone radius is within 6 km, the mean NRSEI value rises faster, and when the buffer zone radius is increased from 6 km to 10 km, the rising trend of the curve tends to flatten out gradually. This indicates that the mine has a greater impact on the ecological environment within 6 km, while the ecological environment is less affected within the buffer zone of 6~10 km.

## 4. Discussion

### 4.1. Advantages of the NRSEI Model

**Figure 12:**
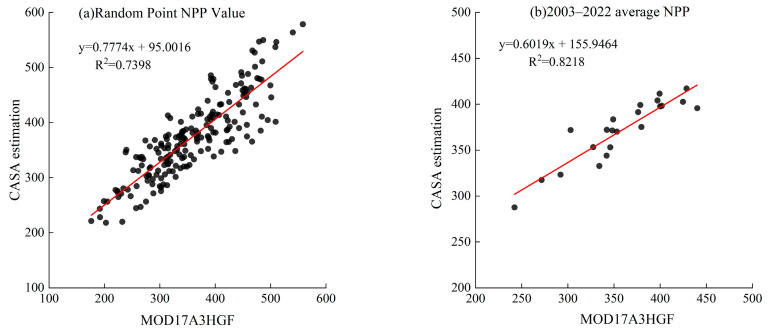
Correlation analysis between CASA estimates and the MOD17A3HGF dataset: (**a**) correlation between CASA model NPP values and MOD17A3 dataset NPP values at randomly sampled points, and (**b**) correlation between CASA model NPP and mean NPP values from the MOD17A3 dataset.

In previous studies, the traditional RSEI was usually adopted in large-scale urban and regional ecological environments due to its representativeness and ease of calculation [48,49]. However, the Shanxi section of the Yellow River Basin is in an arid and semiarid region, and a large number of coal bases are distributed in the region. Unlike other human development activities, the extraction and combustion of mineral resources emits a large amount of carbon, which is difficult to accurately reflect by NDVI alone. The NPP indicator can characterize the carbon sink component and finely characterize regional vegetation destruction and climate change [34]. Therefore, in this paper, the NRSEI-improved remote sensing ecological index model (NRSEI) is constructed based on RSEI by introducing NPP indexes for the characteristics of the Shanxi section of the Yellow River Basin. Unlike previous studies that primarily relied on the MOD17A3HGF dataset, this research employs the CASA model to obtain a more accurate NPP indicator, which is vital for assessing carbon balance and ecological health in the study area. We also conducted a precision verification of the NPP calculated by the CASA model by comparing its estimates with those from the MOD17A3HGF dataset. The results, as shown in Figure 12, indicate that the CASA model provides reliable NPP estimates.

Compared to other advanced remote sensing ecological indices, NRSEI demonstrates its unique advantages. For instance, RSEIFE enhances assessment accuracy by combining ecological functions with remote sensing information, but it has limitations in comprehensively reflecting regional characteristics [32]. MRSEI focuses on multiscale analysis, effectively identifying ecological dynamics, yet it does not perform as well as NRSEI in depicting regional features [50]. IRSEI emphasizes changes in ecological dynamics, while the comprehensiveness of NRSEI gives it an edge in describing the overall ecosystem [33]. ERSEI focuses on effectively identifying ecological environment differences between regions [51]. In contrast, NRSEI captures driving factors of ecological changes more comprehensively by incorporating key indicators like net primary productivity (NPP), thus providing richer ecological information.

### 4.2. Temporal and Spatial Variability of NRSEI Ecological Environment Quality

Although it is difficult to verify the accuracy of the results of the ecological environment quality evaluation with specific data, it can be effectively proved from the relevant conclusions of the statistical yearbook and the literature. During the period of 2003–2022, the overall trend of the NRSEI in the study area was upward, and the ecological environment experienced significant fluctuating changes, which is in line with the results of the previous study on the Shanxi section of the Yellow River Basin [10], indicating the study area’s fluctuation of ecological environment quality. Shanxi Province is the main energy source in the Yellow River Basin, and several nationally planned coal mining areas are also distributed in the Shanxi section of the Yellow River Basin, and the exploitation of coal resources inevitably triggers several ecological safety problems and destroys the ecosystems of soil, water, air, and other ecosystems [12]. The consumption of coal resources in the Yellow River Basin of Shanxi Province is continuously increasing from 2003 to 2022, but the mean value of NRSEI shows an increasing and then decreasing trend, which suggests that other factors inhibit the ecological impacts of mining areas. During this period, afforestation in the Yellow River Basin of Shanxi Province has resulted in an increase in forest cover and a sustained increase in soil and water conservation. This has all improved the ecological environment of the mining area and the whole study area to some extent. In these years, with the government’s emphasis on ecological environment protection, green mining has been realized in some mines, which is very beneficial to the ecological restoration of the mines. Sen + Mann–Kendall analysis verified the spatial heterogeneity of the ecological environment changes in the Shanxi section of the Yellow River Basin, which shows that there is considerable spatial heterogeneity of the ecological environment in the Shanxi section of the Yellow River Basin. The ecological environment in the southeastern part shows a deteriorating trend, with relatively flat terrain and a more concentrated population, in contrast to the northwestern part, which has a complex topography and a smaller population and shows an improving trend. Overall, the results of the Sen + Mann–Kendall analysis are highly consistent with the policy orientation of the spatial pattern in the Shanxi section of the Yellow River Basin.

### 4.3. Uncertainty and Prospects

It is noteworthy that while the improved remote sensing ecological index model NRSEI proposed in this study can relatively objectively reveal changes in ecological environment quality in the Shanxi section of the Yellow River and coal mining areas, several limitations remain. First, the NRSEI model does not adequately consider important factors such as land and water loss and soil erosion caused by mining activities. Additionally, natural factors such as temperature, precipitation, and topography are fundamental variables that significantly influence changes in ecological environment quality. Furthermore, data quality and spatial resolution also have a significant impact on research outcomes. This study utilized the MODIS dataset, but future studies could consider using higher-resolution data to enhance the accuracy and comprehensiveness of ecological assessments. Future research should further explore the comprehensive impacts of these natural and anthropogenic factors on long-term ecological changes.

With the development of remote sensing technology and ecological indicator models, NRSEI has the potential for broader application in ecological environment monitoring and assessment. To enhance its applicability, future research could consider integrating NRSEI with other advanced ecological indices and incorporating more environmental variables for deeper analysis and understanding of ecosystems. This would provide more scientifically based decision support for ecological restoration and sustainable development.

## 5. Conclusions

This study builds on the existing RSEI remote sensing ecological index model. It proposes an improved version, NRSEI, tailored to the Shanxi section of the Yellow River. Using the GEE cloud platform, NRSEI was calculated for the region over the past 20 years. This study analyzed annual average NRSEI trends and spatial distribution for the entire area, coal mining zones, and non-coal mining zones. The following conclusions were drawn:

1. From the perspective of ecological components, the constructed NRSEI effectively integrated the comprehensive information of the five ecological factors of greenness, humidity, dryness, heat, and net primary productivity of vegetation, with an average correlation of more than 0.79. The new remote sensing ecological index model NRSEI constructed in this paper is suitable for the ecological assessment of the Shanxi section of the Yellow River Basin. Compared with the existing remote sensing ecological index model, it can better take into account regional environmental characteristics.
2. From 2003 to 2022, the overall average NRSEI for the Shanxi section of the Yellow River was approximately 0.52, indicating some fluctuation while generally showing an upward trend in ecological environment quality. The ecological environment quality in non-coal mining areas was better than that of the entire study area, while coal mining areas exhibited relatively poorer quality. Furthermore, the trend of change can be divided into four stages, reflecting the dynamic evolution of the ecological environment in this region.
3. Over the past 20 years, the ecological environment of the Shanxi section of the Yellow River and its coal mining areas has undergone significant changes, with significantly more areas showing improvement than deterioration. Notably, the ecological environment in coal mining areas has improved more than in the entire basin. The ecological evolution trend in the northwest region, particularly in the Lvliang Mountain range and the Loess Plateau, has shown a marked increase.
4. The impact of coal mining on the surrounding ecological environment diminishes with increasing distance. Within a 6 km radius, the effects of mining on the ecological environment are most significant. In the 6 to 10 km buffer zone, the influence gradually decreases. This finding provides important reference information for balancing coal mining development and ecological protection.

## Figures and Tables

**Table 1:** Data source description.

Sources	Datasets	Spatial Resolution	Temporal Resolution	Description
GEE	MOD13A1	500 m	16 d	Normalized Difference Vegetation Index (NDVI)
	MOD11A2	1 km	8 d	Land Surface Temperature (LST)
	MOD09A1	500 m	8 d	Normalized Difference Built-up Index (NDBSI) and Wetness (WET)
	MYD09A1	500 m	8 d	Combined with MOD09A1 to extract vegetation, meteorological, and land cover data for Net Primary Productivity (NPP) calculation
	TTERRACLIMATE	4638.3 m	1 m	
	MCD12Q1	500 m	1 a	

**Table 2:** Standard of NRSEI for classification of eco-environmental conditions.

**Level**	**Excellent**	**Good**	**Moderate**	**Fair**	**Poor**
Index	0.8–1	0.6–0.8	0.4–0.6	0.2–0.4	0–0.2

**Table 3:** Classification of ecological quality trends based on β-value and statistic Z.

β	Z	Trend Characteristics	Instructions
β>0.0005	$\left|Z\right|>1.96$	Significant improvement	Passes the 95% significance test
	$\left|Z\right|<1.96$	Minor improvement	Passes the 90% significance test
$\left|\beta \right|\leq 0.0005$	$\left|Z\right|$	Maintain stability	No significant change
β<−0.0005	$\left|Z\right|<1.96$	Mild deterioration	Passes the 90% significance test
	$\left|Z\right|>1.96$	Significant deterioration	Passes the 95% significance test

**Table 4:** The results of PCA (PC1).

Year	NRSEI PC1 Contribution Rate (%)	Year	PC1 Contribution Rate (%)
2003	80.18	2013	72.23
2004	77.54	2014	73.56
2005	82.08	2015	80.36
2006	79.21	2016	72.90
2007	78.61	2017	76.23
2008	79.60	2018	72.39
2009	78.28	2019	74.54
2010	77.92	2020	75.44
2011	73.43	2021	71.69
2012	70.28	2022	70.95

**Table 5:** Test of average correlation of each index.

Years	Average Correlation						Years	Average Correlation					
	NDVI	WET	NPP	NDBSI	LST	NRSEI		NDVI	WET	NPP	NDBSI	LST	NRSEI
2003	0.707	0.714	0.519	0.753	0.651	0.846	2013	0.571	0.354	0.574	0.640	0.555	0.764
2004	0.633	0.414	0.650	0.639	0.586	0.788	2014	0.641	0.420	0.594	0.642	0.566	0.782
2005	0.731	0.545	0.739	0.764	0.743	0.860	2015	0.734	0.559	0.683	0.741	0.687	0.842
2006	0.677	0.389	0.674	0.717	0.649	0.811	2016	0.611	0.389	0.594	0.625	0.590	0.779
2007	0.679	0.418	0.668	0.718	0.649	0.811	2017	0.671	0.497	0.621	0.698	0.644	0.815
2008	0.697	0.511	0.676	0.679	0.638	0.815	2018	0.590	0.404	0.571	0.626	0.592	0.776
2009	0.703	0.506	0.691	0.743	0.689	0.837	2019	0.683	0.507	0.648	0.705	0.663	0.830
2010	0.705	0.514	0.659	0.731	0.685	0.833	2020	0.608	0.440	0.598	0.653	0.634	0.793
2011	0.644	0.411	0.625	0.664	0.577	0.790	2021	0.627	0.448	0.591	0.650	0.563	0.788
2012	0.604	0.418	0.571	0.598	0.507	0.758	2022	0.574	1.511	0.580	0.614	0.574	0.771

Note: The average correlation degree refers to the mean value of the absolute value of the correlation coefficient of each ecological factor, which represents the overall correlation of each ecological factor to NRSEI.

## Data Availability

All the data used in this research are available upon request by e-mail to the corresponding author.
